# Tyrosinase Magnetic Cross-Linked Enzyme Aggregates: Biocatalytic Study in Deep Eutectic Solvent Aqueous Solutions

**DOI:** 10.3390/biom13040643

**Published:** 2023-04-03

**Authors:** Myrto G. Bellou, Michaela Patila, Renia Fotiadou, Konstantinos Spyrou, Feng Yan, Petra Rudolf, Dimitrios P. Gournis, Haralambos Stamatis

**Affiliations:** 1 Biotechnology Laboratory, Department of Biological Applications and Technologies, University of Ioannina, 45110 Ioannina, Greece; myrtobell@gmail.com (M.G.B.); pstm10345@uoi.gr (M.P.); renia.fotiadou@gmail.com (R.F.); 2 Nanomedicine and Nanobiotechnology Research Group, University of Ioannina, 45110 Ioannina, Greece; 3 Ceramics and Composites Laboratory, Department of Materials Science and Engineering, University of Ioannina, 45110 Ioannina, Greece; 4 Zernike Institute for Advanced Materials, University of Groningen, Nijenborgh 4, 9747 AG Groningen, The Netherlands; f.yan.rug@gmail.com (F.Y.);

**Keywords:** biocatalysis, enzyme, tyrosinase, immobilization, deep eutectic solvents, chitosan, caffeic acid, functionalization

## Abstract

In the field of biocatalysis, the implementation of sustainable processes such as enzyme immobilization or employment of environmentally friendly solvents, like Deep Eutectic Solvents (DESs) are of paramount importance. In this work, tyrosinase was extracted from fresh mushrooms and used in a carrier-free immobilization towards the preparation of both non-magnetic and magnetic cross-linked enzyme aggregates (CLEAs). The prepared biocatalyst was characterized and the biocatalytic and structural traits of free tyrosinase and tyrosinase magnetic CLEAs (mCLEAs) were evaluated in numerous DES aqueous solutions. The results showed that the nature and the concentration of the DESs used as co-solvents significantly affected the catalytic activity and stability of tyrosinase, while the immobilization enhanced the activity of the enzyme in comparison with the non-immobilized enzyme up to 3.6-fold. The biocatalyst retained the 100% of its initial activity after storage at −20 °C for 1 year and the 90% of its activity after 5 repeated cycles. Tyrosinase mCLEAs were further applied in the homogeneous modification of chitosan with caffeic acid in the presence of DES. The biocatalyst demonstrated great ability in the functionalization of chitosan with caffeic acid in the presence of 10% *v*/*v* DES [Bet:Gly (1:3)], enhancing the antioxidant activity of the films.

## 1. Introduction

Biocatalysis plays an ever-growing role in the field of chemical transformations and organic synthesis due to the increasing usage of enzymes as catalysts. Enzymes derived from living organisms are employed in catalysis, offering a plethora of advantages compared to chemical catalysts, such as selectivity, biodegradability and non-toxicity [1]. Despite exhibiting these traits, the implementation of enzymes in industrial environments encounters difficulties, as they present low stability and are often not reusable, which enhances the cost. Hence, immobilization of enzymes on various carriers including nanocarriers has emerged, where enzymes are either covalently attached or physically adsorbed on the carrier’s surface or entrapped in it. Enzyme molecules can also cross-link with each other, exhibiting robust biocatalytic performance, high stability and reusability [2,3].

In an attempt to make biocatalysis even more environmentally friendly and sustainable, neoteric solvents, referred as “green” solvents, have gained in attractiveness since they have the potential to replace conventional ones (petrochemical and volatile organic compounds, VOCs) [4,5,6,7]. Among others, the class of “green solvents” consists of ionic liquids (ILs) and deep eutectic solvents (DESs), which share some benign physical characteristics, such as chemical and thermal stability and low vapor pressure [8]. Albeit having in common some physical properties, ILs and DESs differ in their chemistry, and thus they belong to two distinct solvent categories. Specifically, an IL is a mixture of an anion and a cation species that differ in size and form a liquid salt at room temperature. On the other hand, a DES is made by mixing at least two compounds that may or may not contain an ion, namely a hydrogen bond acceptor (HBA) and a hydrogen bond donor (HBD), which interact with each other mainly by hydrogen bonding. Other intra- and inter-molecular interactions take place too and determine each DES’s nanostructure [9].

Compared to ILS, the preparation of DESs is easier and in many cases cheaper, depending on the specific components. Furthermore, DESs are biocompatible, biodegradable, exhibit negligible toxicity, and are energy and atom efficient. DESs can be even more benign when their components derive from nature (amino acids and metabolites) and in this case, they are called natural DESs (NADESs) [9,10,11]. The use of DESs in biocatalysis offers additional advantages, as by choosing the appropriate solvent, a wide range of substrates can be solubilized [5,12]. Furthermore, a DES can act both as reaction medium and as substrate, leading to optimized yields [13,14,15]. For example, *Candida rugosa* lipase has been used for the esterification of (-)-menthol with fatty acids in a solvent-free system, as menthol was the HBA of the DES and each fatty acid was the HBD [16].

Regarding biocatalysis, ILs and DESs have been used as solvents or co-solvents in enzyme reactions and in several cases, it was found that they offer stabilizing effects on the enzymes or enhanced enzyme activities [17,18,19,20,21]. Enzymes catalyzing hydrolysis, esterification, transesterification and oxidation reactions in ILs have been studied thoroughly [20]. On the other hand, the scarce knowledge of the structure-physicochemical properties of many DESs, as well as the lack of understanding of how DESs activate and stabilize the enzymes, call for more systematic research [9,22].

Tyrosinase (EC 1.14.18.1) is an oxidoreductase that catalyzes the hydroxylation of monophenols to *o*-diphenols and the oxidation of *o*-diphenols to *o*-quinones. Tyrosinase is commonly found as a tetramer H_2_L_2_, with the active site situated at the subunit H and containing two Cu ions that participate in catalysis. Tyrosinase is present in a wide variety of organisms; it is found in plants, fungi, mammals and arthropods, among others, and participates in several biological processes [23]. Tyrosinase is of high biotechnological interest as it can be used in the production of L-DOPA, a drug for Parkinson’s disease; in phenol removal from polluted waters; in biosensors; in cosmetics and food industries for the production of phenolic polymers and in the grafting of oxidized phenols onto biopolymers such as chitosan, leading to improved properties [23,24].

Tyrosinase has been immobilized on various carriers and its biocatalytic properties have been studied and compared to those of the free enzyme [25,26,27,28,29]; these studies showed that the immobilized form exhibits enhanced activity and stability. Cross-linking of enzyme molecules is another immobilization technique used for tyrosinase. Typically, in this procedure, agents such as ammonium sulfate or organic solvent are used to precipitate enzyme molecules in an aqueous solution, and then a bio-functional molecule bridge is used for the cross-linking of the neighboring aggregated enzyme molecules, resulting in the formation of cross-linked enzyme aggregates (CLEAs). Glutaraldehyde is a common cross-linker that connects the lysine residues of the enzyme molecules via the formation of a Schiff’s base. This technique is economically viable and simple, as it is a carrier-free sort of enzyme immobilization and there is no need for purified enzymes [30]. Magnetic nanoparticles (maghemite or magnetite) can also be implemented in the immobilization process, giving magnetic properties to the resulting biocatalyst, thus making its reuse more feasible [31]. Recently, tyrosinase from potato peels has been used for the preparation of bio-imprinted magnetic CLEAs, using amino-functionalized magnetic nanoparticles [32]. Either purified or crude tyrosinase from mushrooms has been utilized to prepare tyrosinase CLEAs of high activity and storage stability [33]. In another work, tyrosinase CLEAs have been prepared from fresh mushrooms and successfully used in the treatment of phenol-polluted wastewater [34]. Furthermore, this biocatalyst has been used in the synthesis of L-DOPA from L-tyrosine, displaying higher productivity than those found employing tyrosinase immobilized on other carriers [35].

As far as tyrosinase in neoteric solvents is concerned, only a few works regarding tyrosinase’s catalytic behavior (in free and immobilized form) in ILs have been reported [36,37,38,39,40]. On the other hand, DESs have been implemented in the preparation of tyrosinase CLEAs, resulting in the improved catalytic activity of the immobilized enzyme [41]. To the best of our knowledge, no biocatalytic and structural studies of tyrosinase in DES-based media have been reported.

Chitosan (CS) is a polysaccharide derived from chitin through deacetylation, which is found plentifully in the exoskeletons of crustaceans and insects. This biopolymer exhibits benign properties such as biocompatibility, biodegradability and non-toxicity; for this reason, much research is conducted for its implementation in food, biomedical, pharmaceutical and cosmetic industries [24]. Chitosan possesses low antioxidant activity, which is ascribed to its hydroxyl and amino groups [42] and thus, many approaches have been reported regarding its modification to enhance its antioxidant activity. One method involves the enzymatic functionalization of CS in soluble (homogenous system) or insoluble form (heterogeneous system), with phenolic compounds. In this approach, oxidative enzymes, such as laccases or tyrosinases, catalyze the oxidation of phenolic compounds; the intermediate products can either react with each other or with the initial compound, or be non-enzymatically grafted onto the reactive groups of CS [24]. 

In this work, we report the preparation and characterization of tyrosinase magnetic CLEAs (mCLEAs) and their use in various DES-based media. The effect of the nature and concentration of DESs on the activity, kinetics, stability, and structure of the free and immobilized tyrosinase was investigated. Also, tyrosinase mCLEAs were implemented in the homogeneous modification of the biopolymer chitosan with caffeic acid, in the presence of DES and the antioxidant activity of the resulting modified chitosan film was studied.

## 2. Materials and Methods

### 2.1. Materials

Fresh mushrooms of the genus *Agaricus* were bought from a local market. Glutaraldehyde (25% solution) and methanol (HPLC grade) was bought from Fisher Scientific (Waltham, MA, USA). Ammonium sulfate (99.5%) was purchased from AppliChem (Darmstadt, Germany). Commercial tyrosinase from mushroom (8303 U mg^−1^, solid) was bought from Sigma-Aldrich (St. Louis, MO, USA). DESs’ components were obtained from the reported companies; choline chloride (>98%, ChCl), betaine (98%, Bet), D(-)-Fructose (Fru) from Sigma-Aldrich (St. Louis, MO, USA), ethylammonium chloride (EAC) from Merck (Darmstadt, Germany), choline dihydrogen phosphate (>98%, Chol DHP) from IOLITEC (Heilbronn, Germany), urea (>99%, U) from Fluka (Charlotte, NC, USA), glycerol (0.5% max water, Gly) from Fisher Scientific (Waltham, MA, USA), ethylene glycol (EG) from AppliChem (Darmstadt, Germany), 1,2-Butanediol (>98%, BG) from TCI (Tokyo, Japan) and 1,2-Propanediol (99%, PG) from Acros Organics (Waltham, MA, USA). The water used for the preparation of buffers and the DES ChCl:Fru:H_2_O was double distilled. Iron (II, III) oxide nanoparticles (97%, 50–100 nm), 3,4-Dihydroxy-L-phenylalanine (>98%, L-DOPA), chitosan (75–85% deacetylated), caffeic acid (3,4-Dihydroxycinnamic acid, 97%), acetic acid (>99.8%) and 2,2′-Azino-bis (3-ethylbenzothiazoline-6-sulphonic acid) diammonium salt (98%, ABTS) were bought from Sigma-Aldrich (St. Louis, MO, USA).

### 2.2. Methods

#### 2.2.1. Preparation of Tyrosinase CLEAs and mCLEAs

The tyrosinase CLEAs preparation was performed based on a previous report [38] with some modifications. Fresh mushrooms were weighed (50 g), cut and homogenized in 100 mL of 50 mM cold phosphate buffer pH 7 (4 °C). The mixture was left under magnetic stirring, at 4 °C for 20 min and then centrifuged at 4 °C, 9500× *g* rpm for 10 min. The supernatant was titrated (100 mL) and, only in the case of mCLEAs, 50 mg of iron oxide nanoparticles were added and left under magnetic stirring for 20 min at ambient temperature. Subsequently, the enzyme was precipitated with ammonium sulfate (60%), by slowly adding the appropriate amount of salt under magnetic stirring. Afterward, glutaraldehyde solution was slowly added to reach the final concentration of 2% *v*/*v* and the mixture was left under magnetic stirring for 3 h at ambient temperature. Then, the mixture was centrifuged at 9500× *g* rpm, 4 °C for 10 min and washed with buffer until the supernatant was clear and colorless. Τhe supernatant and the washings were collected and checked for enzyme activity and no activity was detected, which indicates that tyrosinase molecules was incorporated in the CLEAs. The precipitant (CLEAs/mCLEAs) was dried under vacuum and then ground in a mortar with a pestle. The biocatalysts were stored at −20 °C.

#### 2.2.2. Characterization of Tyrosinase CLEAs/mCLEAs

Tyrosinase CLEAs and mCLEAs were analyzed by Fourier-transform infrared spectroscopy (FTIR), X-ray diffraction (XRD) and X-ray photoelectron spectroscopy (XPS).

The FTIR spectroscopy measurements of magnetic iron oxide nanoparticles, tyrosinase CLEAs and mCLEAs were performed with a Jasco FT/IR 4700 spectrometer (JASCO, Tokyo, Japan), equipped with a Peltier stabilized DLaTGS detector. Samples were prepared as KBr pellets (3% *w*/*w* of samples in KBr); spectra were acquired in the range 400–4000 cm^−1^, averaging 64 scans collected with 4 cm^−1^ resolution.

XPS data were collected using a Surface Science Instruments SSX-100 ESCA instrument (SSI, Mountain View, CA, USA) equipped with a monochromatic Al K_α_ X-ray source (hν = 1486.6 eV). The samples were dispersed in chloroform by sonication for 10 min, and a small drop of the suspension was left to dry in air on a homemade 150 nm thick gold film supported on mica [43]. During the measurement, the pressure was kept below 1.0 × 10^−9^ mbar in the analysis chamber; the electron take-off angle with respect to the surface normal was 37°. A flood gun was used during the XPS measurements to compensate for charging effects. The XPS data were acquired on a spot with a diameter of 1000 µm and the energy resolution was set to 1.26 eV (or 1.67 eV for a survey scan). Binding energies (BE) are reported as ±0.1 eV when deduced from a fit and referenced to the C1*s* photoemission peak centered at a binding energy of 284.6 eV. All spectra were analyzed using the least squares fitting program Winspec (LISE laboratory, University of Namur, Belgium). Deconvolution of the spectra included a Shirley baseline subtraction, fitting with a minimum number of peaks consistent with the structure of the surface, taking into account the experimental resolution. The peak profile was taken as a convolution of Gaussian and Lorentzian functions. All measurements were conducted on freshly prepared samples, and three different spots were measured on each sample to check for homogeneity.

XRD patterns were recorded using a D8 ADVANCE diffractometer (Bruker AXS, Madison, WI, USA) with a Cu K_α_ radiation (wavelength of 1.5406 Å) and a secondary beam graphite monochromator. The patterns were recorded from 2–80° 2*θ* at a constant rate of 0.01° s^−1^.

#### 2.2.3. Preparation of DESs

The DESs that were used in this work are presented in Table 1 and were prepared as reported elsewhere [44], with slight modifications. Briefly, specific amounts of HBAs and HBDs corresponding to their molar ratio were weighted and put in a glass vial. The DESs that contained glycerol or fructose were incubated at 80 °C, whereas the other DESs were incubated at 100 °C until a clear solution was formed. During the incubation, the mixtures were vortexed every 10 min. DESs were then stored at 30 °C.

#### 2.2.4. Activity Assays in DES Aqueous Solutions

Free tyrosinase and tyrosinase mCLEAs/CLEAs activities were determined by using L-DOPA as the model substrate and its oxidation to dopachrome as the model reaction. For free tyrosinase, 4 μg mL^−1^ of the enzyme was added in 50 mM phosphate buffer pH 7 containing DESs at the concentrations of, 10%, 20%, 50% and 70% *v*/*v* and L-DOPA at the final concentration of 2 mM. The reaction was observed spectrophotometrically with a microplate reader (Infinite M200 Pro, Tecan, Männedorf, Switzerland) at 492 nm at 30 °C. Measurements were conducted in triplicate. Experiments in DES-free solutions were also conducted and used as control samples.

In the case of tyrosinase mCLEAs, 1 mg mL^−1^ of the biocatalyst was added in buffer or aqueous DES solutions (10%, 20%, 50% and 70% *v*/*v*) containing 10 mM L-DOPA and the reaction mixture was put at a thermoshaker at 700 rpm and 30 °C. At 0 min and every 2 min for a total time of 10 min, aliquots of the solution were withdrawn by separating the biocatalyst from the solution with the use of a magnet, measured at 492 nm and returned in the reaction mixture, in order to determine the initial rate of the reaction. Regarding tyrosinase CLEAs, the activity was measured as referred for mCLEAs, in buffer (0% *v*/*v* DES). The activity of tyrosinase CLEAs was 2.4 U/g, whereas the activity of tyrosinase mCLEAs was 12.2 U/g, where 1 U (μmol min^−1^) is defined as the amount of biocatalyst that catalyzes the production of 1 μmol dopachrome when assayed in 1 mL phosphate buffer (pH 7, 50 mM), containing 10 mM L-DOPA, at 30 °C and 700 rpm. Measurements were conducted in triplicate.

#### 2.2.5. Kinetic Studies

To determine the apparent kinetic constants of tyrosinase mCLEAs, namely the apparent Michaelis-Menten constant (K_m_^app^) and the apparent maximum reaction rate (V_max_^app^), the biocatalyst’s activity was measured in various concentrations of L-DOPA (0.5–23 mM) in 10% *v*/*v* of numerous DESs. DESs with the same HBA (ChCl) and different HBDs, as well as with the same HBD (Gly) and different HBAs were selected, in order to investigate the effect that the HBA or the HBD of each DES may have on the activity of the biocatalyst. The activity measurement was performed as described in Section 2.2.4. and the activity was expressed as μM min^−1^, by implementing the Beer-Lambert Law. The extinction coefficient of dopachrome used was 3700 M^−1^ cm^−1^ [45]. The kinetic constants were computed from nonlinear regression fitting of the initial reaction rates corresponding to the L-DOPA concentrations, applying the Michaelis-Menten model (EnzFitter, Biosoft, Cambridge, UK). Measurements were conducted in triplicate.

#### 2.2.6. Stability of Free Tyrosinase/Tyrosinase mCLEAs in DESs

To investigate the impact of the DESs on tyrosinase’s stability, the activities of free tyrosinase (5 μg mL^−1^) and tyrosinase mCLEAs (1 mg mL^−1^) were first measured in buffer and then pre-incubated in DES-free solution and 10% *v*/*v* of all DESs, at 30 °C, 700 rpm, for 24 h. In the case of free tyrosinase, after the incubation, specific amount of the enzyme was added to the buffer containing L-DOPA and the reaction was monitored spectrophotometrically as described in Section 2.2.4. Regarding mCLEAs, after incubation, the biocatalyst was washed three times with buffer and its activity was measured in DES-free solution, as described in Section 2.2.4. Measurements were conducted in triplicate. The relative activity (%) was defined as follows:

Relative activity (%) = Activity after incubation in x% *v*/*v* DES × 100/Activity after incubation in DES-free solution (1)

#### 2.2.7. Reusability of Tyrosinase mCLEAs 

The reusability of tyrosinase mCLEAs in the oxidation of L-DOPA in buffer was evaluated for 10 consecutive cycles. The concentration of the biocatalyst used was 1 mg mL^−1^ and the activity assay was conducted as described in Section 2.2.4. At the end of each cycle, tyrosinase mCLEAs were recovered by applying a magnetic field and washed thrice with 50 mM phosphate buffer at pH 7. Measurements were conducted in triplicate. The relative activity (%) was defined as follows:

Relative activity (%) = Remaining activity after each cycle × 100/Activity after the first cycle(2)

#### 2.2.8. Storage Stability of Tyrosinase mCLEAs

The storage stability test for tyrosinase mCLEAs was performed for up to one year. The biocatalyst was stored at −20 °C and its residual activity was measured after 5, 7, 9 and 12 months, as detailed in Section 2.2.4. Measurements were conducted in triplicate.

#### 2.2.9. Fluorescence Study

To find out whether the presence of DESs causes any changes in the conformation of tyrosinase, fluorescence spectra of the free enzyme were recorded on a luminescence spectrofluorometer Jasco-8300 (JASCO, Tokyo, Japan). Solutions of tyrosinase (10 μg mL^−1^) in 0.5 mM phosphate buffer pH 6.8 containing 10%, 20% and 50% *v*/*v* of various DESs were prepared and left for 10 min before the fluorescence measurement in order for the enzyme to take its final conformation. The excitation wavelength was set to 280 nm and a 1.0 cm fluorescence quartz cuvette was used. The emission spectra were recorded in the range 300–450 nm with the slit width set to 5 nm, the scanning speed set to 100 nm min^−1^ and summing 2 scans. The spectra of blank solutions of buffer containing the different concentrations of DESs without the enzyme were used as baseline.

#### 2.2.10. Circular Dichroism Analysis

Circular dichroism (CD) measurements were conducted on a Jasco J-1500 Circular Dichroism Spectrophotometer (JASCO, Tokyo, Japan) equipped with a Peltier temperature control system. The tyrosinase concentration was 0.012 mg mL^−1^ (pure) in 10 mM phosphate buffer pH 6.8 and the concentration of the tested DESs was 10% *v*/*v*. The spectra were recorded after a 5 min or a 24 h- incubation of tyrosinase in the DES-buffer solutions. The optical path length of the quartz cell was 1 cm and the temperature was set to 25 °C. All spectra were recorded in the far ultraviolet (200–260 nm), at a scan speed of 50 nm min^−1^, 2 nm bandwidth, 1 s D.I.T and summing 2 scans. For each sample, a baseline was recorded and subtracted from the enzyme spectrum. The secondary structure element content was estimated by implementing the K2D algorithm on the DichroWeb server [46].

#### 2.2.11. Homogeneous Enzymatic Modification of Chitosan with Caffeic Acid

For the tyrosinase mCLEAs-mediated homogeneous modification of chitosan, a 1.5% *w*/*v* solution of chitosan (CS) in 1% *v*/*v* acetic acid/ddH_2_O was prepared and incubated at 70 °C for 18 h, in order for chitosan to become well-dissolved. Afterward, the solution was centrifuged at 9500× *g* rpm for 15 min to remove any undissolved chitosan. Then the pH of the CS solution was adjusted to 5.8 and it was stored at 4 °C. For the modification of CS with caffeic acid, 5 mg mL^−1^ mCLEAs and 1 mL of caffeic acid dissolved in methanol (final concentration 2 mM) were added in 9 mL of the CS solution and the reaction mixture was incubated at 30 °C and 130 rpm for 30 min. Thereafter, mCLEAs were removed by applying an external magnetic field, the solution was poured in a petri dish of 5 cm diameter and left to dry at 30 °C. For the homogeneous modification of CS in the presence of DES, a 10% *v*/*v* Bet:Gly (1:3)/CS solution was prepared and left under magnetic stirring for 1 h for the homogenization of the mixture before adding the substrate and the enzyme, as described. Dried films were rinsed trice with methanol to remove unbound caffeic acid and left to dry again before use. Dried films were stored at 4 °C.

#### 2.2.12. Characterization of Chitosan Films

Attenuated total reflectance (ATR)–Fourier-transform infrared (FTIR) spectroscopy was used for the characterization of the unmodified and modified CS films. ATR spectra were recorded on a Jasco FT/IR-4700 (JASCO, Tokyo, Japan) infrared spectrometer with the ATR PRO ONE accessory. All spectra were acquired in the range of 400–4000 cm^−1^, averaging over 128 scans, collected with a resolution of 2 cm^−1^.

#### 2.2.13. Antioxidant Activity of Chitosan Films

The antioxidant activity of the chitosan films was examined as described elsewhere [47], with minor modifications. A solution of ABTS^•+^ free radical was prepared by mixing 7 mm ABTS and 2.45 mM potassium persulphate in ddH_2_O and left for 18h in the dark at 25 °C. Just before use, the ABTS^•+^ solution was diluted with phosphate buffer saline (PBS) until its absorbance at 734 nm reached 0.700 ± 0.025. Then, pieces of CS films with specific dimensions (5 mm × 3 mm) were added in 1 mL of the ABTS^•+^ solution. At 0, 5, 10, 20, 30, 40 and 60 min, 300 μL were withdrawn and the absorbance was measured at 734 nm with a microplate reader (Infinite M200 Pro, Tecan, Männedorf, Switzerland). The following equation was used in order to determine the antioxidant activity (%):

Antioxidant activity (%) = (A_control_ − A_sample_) × 100/A_control_ (3)

where A_control_ is the initial absorbance of the ABTS^•+^ and A_sample_ is the absorbance of the remaining ABTS^•+^ in the presence of the sample. The experiment was conducted thrice, and the absorbance of the blank sample ABTS^•+^ was also measured.

## 3. Results and Discussion

### 3.1. Preparation and Characterization of Tyrosinase CLEAs/mCLEAS

In the present work, tyrosinase was extracted from fresh mushrooms and used in a carrier-free immobilization method for the preparation of CLEAs, where the enzyme molecules are cross-linked via covalent bonding through glutaraldehyde coupling chemistry. For the first time, magnetic nanoparticles (iron (II, III) oxide) were implemented in the CLEAs preparation, and the biocatalyst was characterized through various spectroscopic techniques, such as FTIR, XPS and XRD.

**Figure 1:**
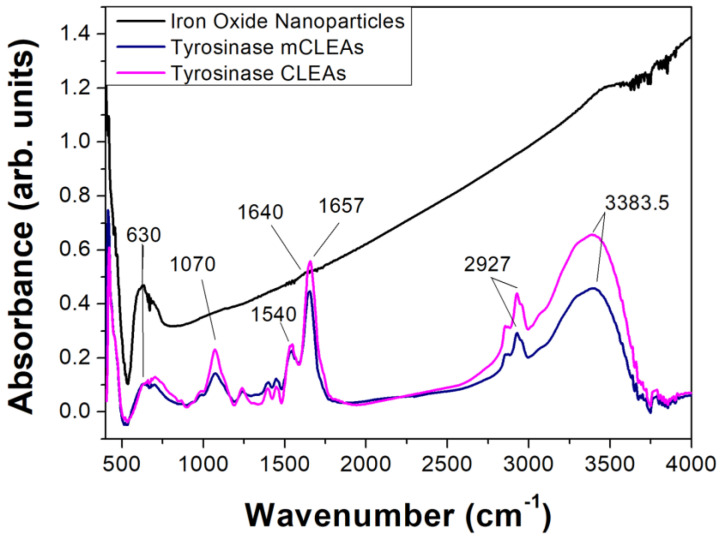
FTIR spectra of magnetic nanoparticles, tyrosinase CLEAs and tyrosinase mCLEAs.

The FTIR spectra of magnetic nanoparticles, tyrosinase CLEAs and tyrosinase mCLEAs are presented in Figure 1. Regarding the magnetic nanoparticles, the absorption band at 630 cm^−1^ corresponds to the stretching vibration of the Fe-O bond [48,49]. The same band was also clearly observed in the case of tyrosinase mCLEAs, whereas in the case of non-magnetic CLEAS no distinct peak was found at this wavenumber. However, the absorption appearing in this region may be ascribed to bond vibrations of other organic compounds found in the mushroom extract from which tyrosinase CLEAs and mCLEAs were prepared [50]. In the FTIR spectra of tyrosinase CLEAs and mCLEAs, the enzymes’ characteristic absorption bands known as Amide I (peaks at 1654 and 1652 cm^−1^), Amide II (1540 cm^−1^) and Amide III (peaks in the range of 1250–1350 cm^−1^) are observed for both biocatalytic systems. These bands are mostly associated with the C=O stretching vibration (Amide I), the N-H bending vibration and the C-N stretching vibration (Amide II and Amide III) [26]. FTIR analysis has also been used by other researchers with the aim of proving the successful preparation of cellulase CLEAs and cellulase magnetic CLEAs, by observing similar bands [51].

**Figure 2:**
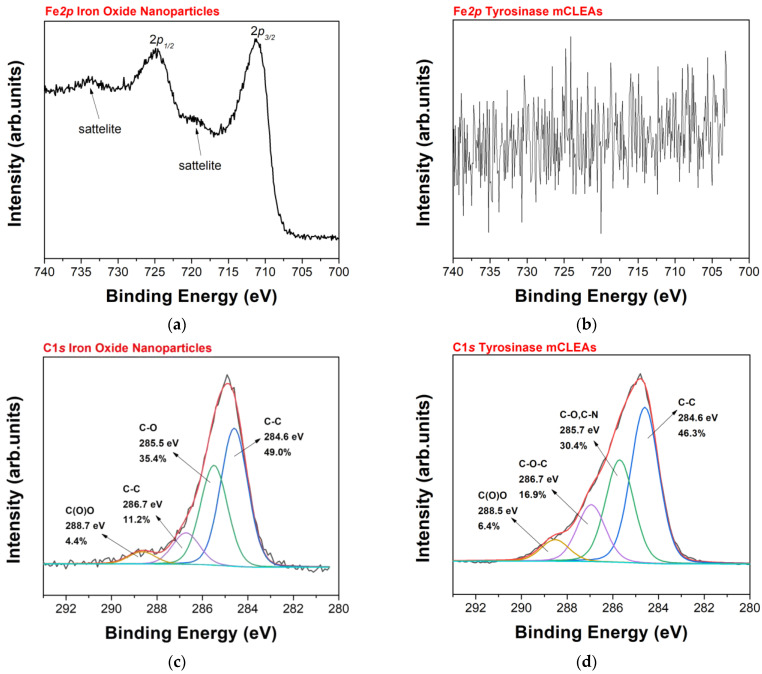
XPS spectra of the Fe*2p* core level region of (**a**) iron oxide nanoparticles; (**b**) tyrosinase mCLEAs and XPS spectra of the C1*s* core level region of (**c**) iron oxide nanoparticles; (**d**) tyrosinase mCLEAs.

To distinguish the magnetic phases of iron oxide nanoparticles, XPS was employed. In the Fe-*2p* core level spectrum shown in Figure 2a, the presence of satellite peaks at ~719.0 eV for the 2*p_3/2_* component and at ∼733.0 eV for the 2*p_1/2_* (marked with arrows) and characteristic for *γ*-Fe_2_O_3_ [52,53], demonstrates the existence of both *γ*-Fe_2_O_3_ and Fe_3_O_4_ magnetic structures. For the pure magnetite phase, these characteristic satellite peaks [54] are in fact absent. From the C1*s* core level spectrum, we receive information for the carbon functionalities covering the iron oxide nanoparticles such as C-C, C-O, C-O-C and C(O)O at 284.6 eV, 285.5 eV, 286.7 eV and 288.7 eV, respectively, as presented in Figure 2. 

XPS is a surface sensitive spectroscopic technique that can retrieve information from a probing depth of approximately 10 nm. The fact that we cannot detect any Fe in the spectrum in Figure 2b leads to the conclusion that the protein molecules have covered the Fe nanoparticles with a thickness of at least 10 nm. For this reason, the C1*s* spectrum in Figure 2d refers to the enzyme structure and the various components can be attributed to C-C (46.3% relative intensity), C-O, C-N (30.4%), C-O-C (16.9%), and carboxyl bonds (6.4%).

**Figure 3:**
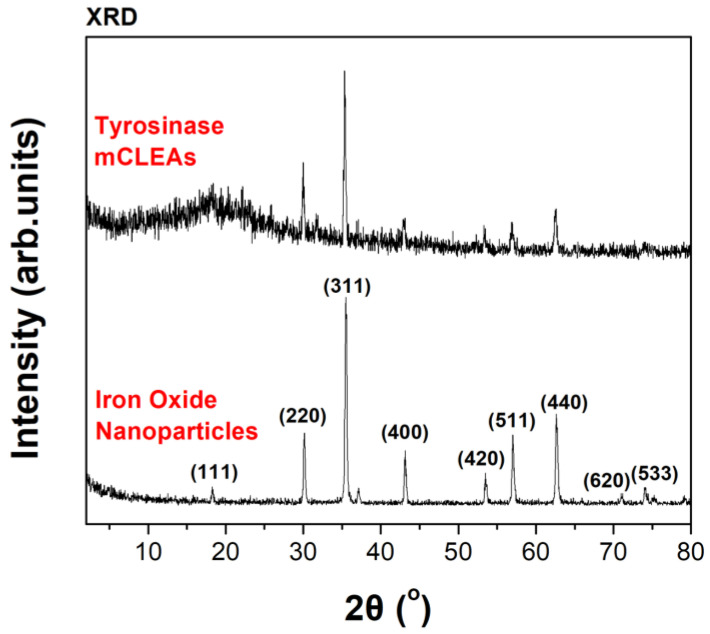
XRD patterns of iron oxide nanoparticles and tyrosinase mCLEAs.

Figure 3 displays the X-ray powder diffractograms of the iron oxide nanoparticles alone and the oxide nanoparticles with the enzyme molecules (tyrosinase mCLEAs) in order to investigate their crystalline structure. One distinguishes the (220), (311), (400), (420), (511), and (440) diffraction peaks, which are in good agreement with the literature and the spinel structure. From the XRD, the diffraction peaks are similar to the structure of magnetite (JCPDS card. No 74-0748). However, due to the similarity of magnetite and maghemite in XRD, we employed XPS to distinguish the Fe^+2^ and Fe^+3^ phases. The XRD pattern of tyrosinase mCLEAs is similar to the one of iron oxide nanoparticles demonstrating that functionalization with the enzyme does not affect the core of magnetite/maghemite. The noisy background and the overly broad peak between 15° and 25° for iron oxide nanoparticles derive from the amorphous organic matter of the biocatalyst testifying to the successful functionalization of the iron oxide nanoparticles.

### 3.2. Biocatalytic Characteristics of Free and Immobilized Tyrosinase in DES Aqueous Solutions

The biocatalytic activity of the prepared tyrosinase CLEAs was determined using the oxidation of L-DOPA as model reaction. It is interesting to note that comparing non-magnetic and magnetic CLEAs, the latter exhibited a 5 times higher oxidation activity (Figure S1a). Hence, because of their greater activity and their easier recovery by merely utilizing a magnet (Figure S1b), tyrosinase mCLEAs were used throughout this study.

#### 3.2.1. Reusability and Storage Stability of Tyrosinase mCLEAs

**Figure 4:**
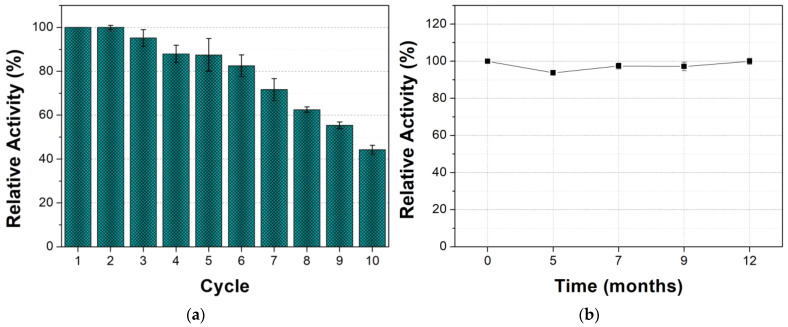
(**a**) Reusability of tyrosinase mCLEAs in buffer: relative activity for 10 consecutive cycles; (**b**) storage stability of tyrosinase mCLEAs: relative activity after storing for different times at −20 °C. As 100% is defined the enzyme activity at the beginning of the test (initial activity). The reaction conditions were 1 mg mL^−1^ of biocatalyst and 10 mM L-DOPA in 50 mM phosphate buffer pH 7, at 700 rpm and 30 °C.

The ability of an immobilized biocatalyst to be repeatedly used is of paramount importance for its use in large-scale applications. The results regarding the reusability of tyrosinase mCLEAs are presented in Figure 4a. The biocatalyst retained ~90% of its initial activity after 5 cycles of use and ~45% after 10 cycles. These results were similar or better than those presented in other works on tyrosinase immobilized on different carriers [25,29], and comparable to those reported for other tyrosinase CLEAs preparation [34].

As demonstrated in Figure 4b, tyrosinase mCLEAs showed excellent storage stability at −20 °C, as no loss of activity was observed after a period of one year. This outcome indicates that this biocatalyst can be stored at −20 °C for further use in any application after a long period of time. This result is superior to those observed for different types of immobilized tyrosinase stored at 4 °C [25,29,33].

#### 3.2.2. Effect of DES Nature and Concentration on the Tyrosinase Activity

**Figure 5:**
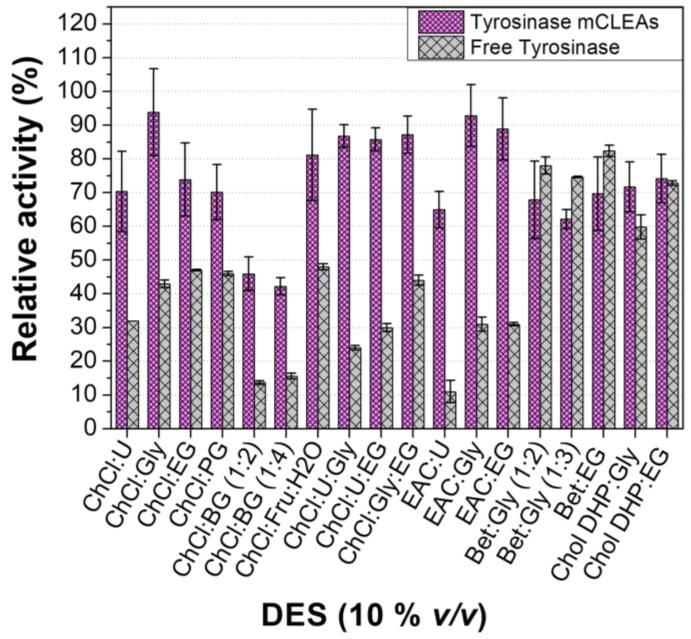
Relative activity of tyrosinase mCLEAs and free tyrosinase in 10% *v*/*v* aqueous solutions of certain DESs. 100% is defined as the activity of tyrosinase mCLEAs or free tyrosinase in DES-free solution.

The activities of free tyrosinase and tyrosinase mCLEAs in 10% *v*/*v* of various DESs are presented in Figure 5. As can be seen, the enzymatic activity of both immobilized and free tyrosinase strongly depends on the nature of the HBA and HBD used for the formation of the various DESs. In almost all DESs used, the relative oxidation activity of the immobilized tyrosinase was significantly higher than that of the free enzyme. An exception was observed in the DESs formed with betaine and choline dihydrogen phosphate, where the relative activities of free and immobilized tyrosinase are similar. The high activity of free tyrosinase in these DESs is in accordance with what has been observed for another oxidase, such as laccase from *Bacillus HR03*, where the activity of free enzyme was higher in Bet-based DES than in ChCl-based DESs, and this was attributed to the presence of the chloride anion, which inhibited the catalytic activity of the free laccase [55]. This inhibitory effect of the Cl anion has also been reported for tyrosinase in a single study [56]. Furthermore, the replacement of ChCl with Chol DHP led to an improvement in free tyrosinase activity, which is in accordance with what has been observed for laccase [57]. 

Independently of the HBA used, the highest activity of tyrosinase mCLEAs was observed in the DESs formed with glycerol (Gly) or ethylene glycol (EG), pointing to the crucial role of the HBD used. In the case of the DES formed with glycerol, the oxidation activity of tyrosinase mCLEAs was around 90% of that in the buffer alone, which is significantly higher than in media containing 5% of various ionic liquids [38]. 

Based on the above results, we can conclude that immobilized tyrosinase preserves its activity in the presence of most DESs (10% *v*/*v*) to a greater extent than the free enzyme, while the nature of each DES, which depends on both the HBA and the HBD, crucially influences the free and immobilized enzyme’s activity.

**Figure 6:**
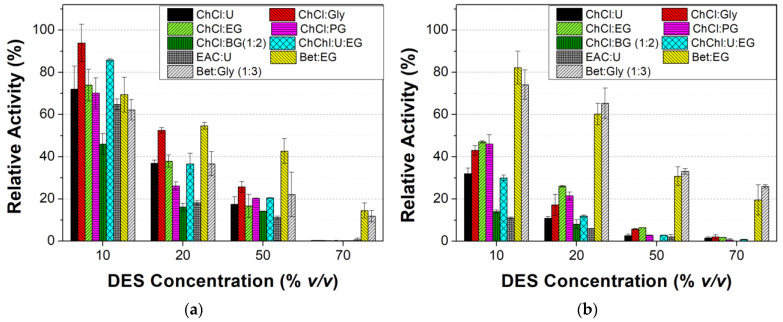
Relative activity of (**a**) tyrosinase mCLEAS and (**b**) free tyrosinase at increasing concentrations of certain DESs. 100% is defined as the activity of tyrosinase mCLEAs or free tyrosinase in DES-free solution.

Figure 6 shows the effect of the DES concentration in various DES-based media on the activity of free and immobilized tyrosinase. As can be seen, in most cases studied, an increase in the DES concentration leads to a significant decrease in the enzyme activity for both enzyme forms. An enzyme activity decrease when the DES concentration increases have also been observed for laccase [57], as well as for tyrosinase in various ionic liquid-based media [36]. This behavior could be attributed to the effect of the DES components on the enzyme structure as well as to the high viscosity of the DES-based reaction media [58]. The enhanced viscosity of high-content DES-based media could increase the mass transfer limitations of the substrates to the tyrosinase microenvironment, which would be expected to reduce its oxidase activity [59]. It must be noted that even at high concentrations of betaine-based DESs the enzyme retains a remarkable part of its initial activity which leads to the inference that betaine-based DESs offer a stabilizing effect to tyrosinase. The stabilizing effect of betaine-based DESs over ChCl-based ones has also been reported for laccase [55]. Additionally, the protective effect of the immobilization on the ability of tyrosinase to retain its catalytic activity was confirmed because, contrary to free tyrosinase, for tyrosinase mCLEAs the relative activity at increased concentrations of almost all DESs was higher.

#### 3.2.3. Kinetic Study of Tyrosinase mCLEAs in the Presence of Various DESs

To further examine the effect of the presence of DESs (10% *v*/*v*) on the catalytic activity of tyrosinase mCLEAs, the apparent Michaelis-Menten constant (K_m_^app^) and the apparent maximum reaction rate (V_max_^app^) were determined using the Michaelis—Menten kinetic model. The apparent kinetic parameters of tyrosinase mCLEAs in DESs with the same HBD (Gly) are presented in Table 2 and the parameters in DESs with the same HBA (ChCl) are presented in Table 3. 

As can be seen, the nature of HBDs and HBAs affects the values of the apparent kinetic parameters. In all cases, the K_m_^app^ values in DES-based media were higher than those observed in buffer except in the case of Bet:Gly (1:3), where the K_m_^app^ value was lower. On the other hand, a decrease in the V_max_^app^ values was observed for almost all DESs independently of the nature of HBD and HBA. An exception was observed for Chol DHP:Gly in which the enzyme presented a higher V_max_^app^ value than that observed in the presence of ChCl:Gly and in buffer alone. This may be ascribed both to the absence of the Cl anion, which may inhibit the enzyme activity [56], and the presence of the dihydrogen phosphate ion, which may affect positively the enzyme activity.

Hence, one can conclude that both HBAs and HBDs reduce the catalytic activity because they affect the affinity of the enzyme for the substrate (maintained or lower K_m_ values). As indicated before, this behavior could be attributed to the effect of the DES components on the enzyme structure, as well as to the fact that the nature of each DES causes changes in some physicochemical properties, such as polarity and viscosity of the reaction medium, which in turn could affect the enzyme activity and/or the ability of the substrate to reach the active site of the enzyme [55,60,61,62].

#### 3.2.4. Effect of DESs on the Stability of Tyrosinase

**Figure 7:**
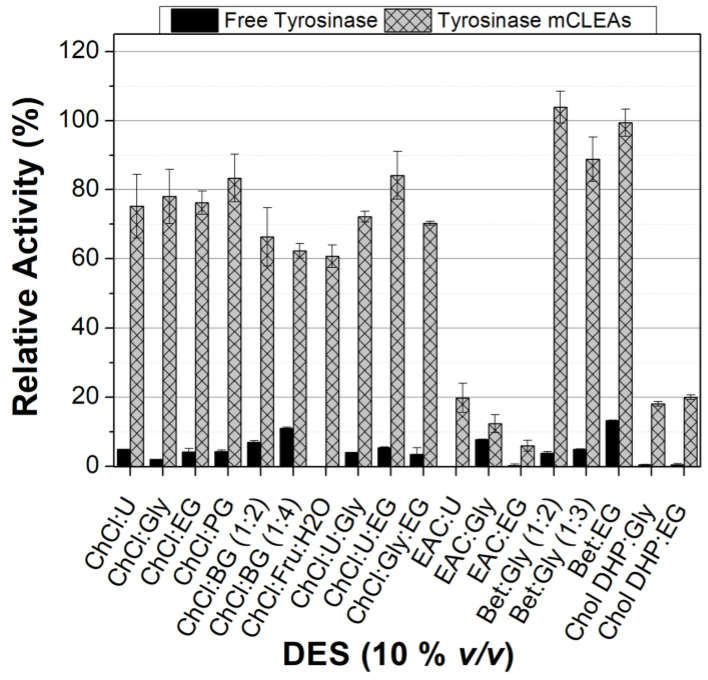
Relative activity of free tyrosinase and tyrosinase mCLEAs in 10% *v*/*v* of various DESs after pre-incubation at 30 °C for 24 h. 100% is defined as the residual activity of free tyrosinase and tyrosinase mCLEAs after pre-incubation at 30 °C for 24 h in DES-free solution.

The stability of free tyrosinase and tyrosinase mCLEAs was further studied by measuring the residual activity after pre-incubation of the biocatalyst in 10% *v*/*v* of various DESs at 30 °C. As seen in Figure 7, for all DES-based media used, the relative activities of tyrosinase mCLEAs are significantly higher than those of the free enzyme. It is noteworthy that the relative activity of tyrosinase mCLEAs strongly depends on the nature of the DES used, in accordance with reports for other enzymes [59,63]. The highest relative activity of the immobilized tyrosinase was observed in betaine-based DESs for all the HBDs used which agrees with the results obtained for the tyrosinase oxidation activity in increasing concentrations of DESs (Figure 6). The positive effect of some betaine-based DES media was also recently reported for an immobilized *β*-glucosidase [64].

### 3.3. Structural Studies of Free Tyrosinase

#### 3.3.1. Fluorescence of Free Tyrosinase in DES-Buffer Solutions

To investigate the impact of certain DESs on the tyrosinase structure, the effect of increased concentrations (10, 20, 50% *v*/*v*) of various DESs on the fluorescence of free tyrosinase was determined. It is well established that observed alterations in the fluorescence emission intensity or shifts in the emission wavelength of a protein denote changes in the global protein structure [65]. 

**Figure 8:**
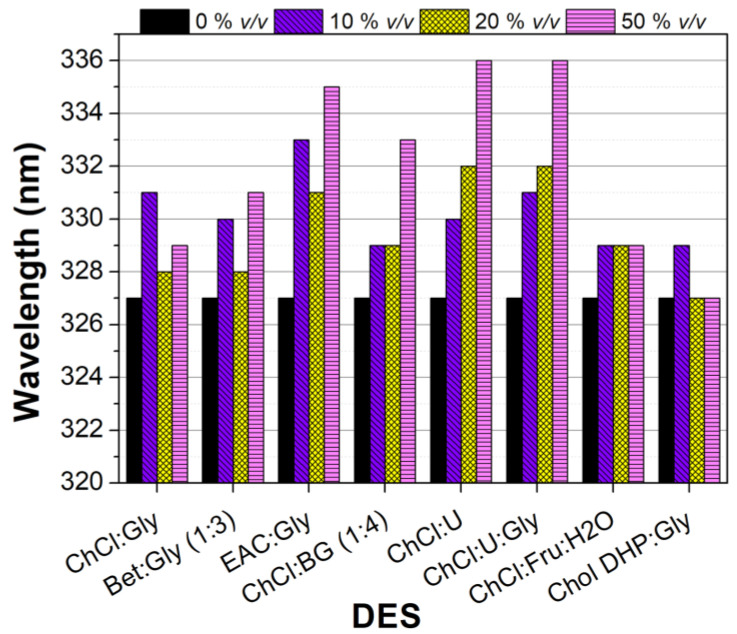
Maximum emission wavelength (λ_max_) of free tyrosinase’s fluorescence emission spectra, for increasing concentrations of various DESs.

The fluorescence emission spectra of free tyrosinase are presented in Figure S2 and the observed shifts in λ_max_ for every concentration of each DES are presented in Figure 8. In most cases, a red shift of the emission maximum was observed when specific amounts of the DESs were added to the tyrosinase aqueous solution. Especially, greater red shifts (λ_max_ changed up to 9 nm) were noted when EAC:Gly, ChCl:U, ChCl:U:Gly and ChCl:BG (1:4) were used at the concentration of 50% *v*/*v*. This red shift of the λ_max_ indicates that the enzyme has adopted a more relaxed structure, where the environment of the aromatic amino acids has become more polar as these amino acids have become more exposed to the solvent structure [65]. These structural alterations that are observed in DES-based media could reduce the oxidation activity of tyrosinase, which is in accordance with the results described before (Figure 6).

Apart from the shift in λ_max_, changes in the intensity of the fluorescence emission were observed and were most pronounced in the case of Chol DHP:Gly and Bet:Gly (1:3); this points to general changes in the microenvironment of the aromatic amino acids [60].

#### 3.3.2. Circular Dichroism of Tyrosinase in the Presence of DESs

CD spectra were measured in order to further investigate the effect of various DESs (10% *v*/*v*) on the secondary structure of free tyrosinase. The results regarding the secondary structure element content of tyrosinase after incubation in DES solutions for 5 min or 24 h are presented in Table 4 and Table 5, respectively, and the CD spectra are shown in Figure S3. The conformation of tyrosinase was marginally affected after 5 min of incubation where a slight decrease in the α-helical and an increase in the *β*-sheet content in most cases were noted. After 24 h of incubation dramatic changes were observed, namely a more important reduction in the α-helix content and a corresponding increase in the *β*-sheet content. These conformational changes have been associated with the unfolding of the tyrosinase’s polypeptide chain and thus, the adoption of a looser enzymatic structure [66,67,68,69,70]. The diminished rigidity of free tyrosinase in the presence of 10% *v*/*v* DESs is consistent with the results obtained from the fluorescence study and agrees with the reduced catalytic activity and stability that were observed (Figure 5 and Figure 7).

### 3.4. Functionalization of Chitosan with Caffeic Acid in the Presence of DES

**Figure 9:**
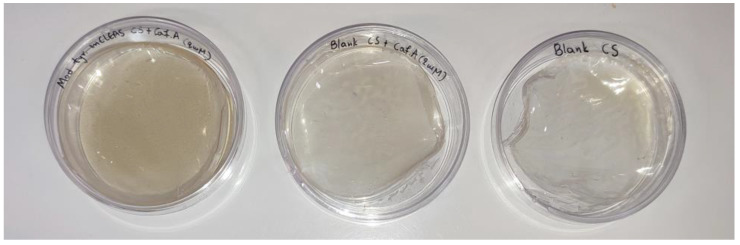
Photographs of a mCLEAs-modified chitosan film with caffeic acid (**left**), a blank chitosan film with caffeic acid (**middle**) and a neat chitosan film (**right**).

Tyrosinase mCLEAs were used in the enzymatic functionalization of CS via oxidation-grafting reaction of caffeic acid (CA) derivatives, in the presence of 10% *v*/*v* Bet:Gly (1:3). This DES was selected as plasticizer of the CS film due to the decent activity and the excellent stability that tyrosinase mCLEAs showed. The employment of DESs in CS films results in a considerable decline in the rigidity of the films; for instance, the addition of various acidic DESs or ChCl:Gly to a CS solution enhanced the plasticity of the obtained films and altered their physicochemical and mechanical properties and their ionic conductivity [71,72,73,74,75,76]. To the best of our knowledge, this is the first time that Bet:Gly is used as a plasticizer for CS films. When tyrosinase mCLEAs were added to the reaction mixture that contained the solution of CS, DES and CA, gradual gelation of the solution was observed (Figure S4). The reaction was stopped by removing the biocatalyst after 30 min of incubation and the solution was left to dry until a film formed (Figure 9). Liu et al. [77] demonstrated that tyrosinase-catalyzed oxidation of caffeic acid in a chitosan solution results in gelation, which is attributed to a possible covalent crosslinking of the oxidized products on chitosan. In addition, the change of color of CS during the oxidation-grafting reaction of phenolics, as it is observed in our case (Figure S5), has been associated with the grafting of the reaction products onto the polymer [78]. No browning and gelation were noted in the solutions without the enzyme, indicating that no grafting or crosslinking of CA derivatives took place. Hence, the brownish color of the modified films and the apparent phenomenon of gelation that are observed in the present work suggest the successful modification of CS in the presence of 10% *v*/*v* Bet:Gly (1:3).

**Figure 10:**
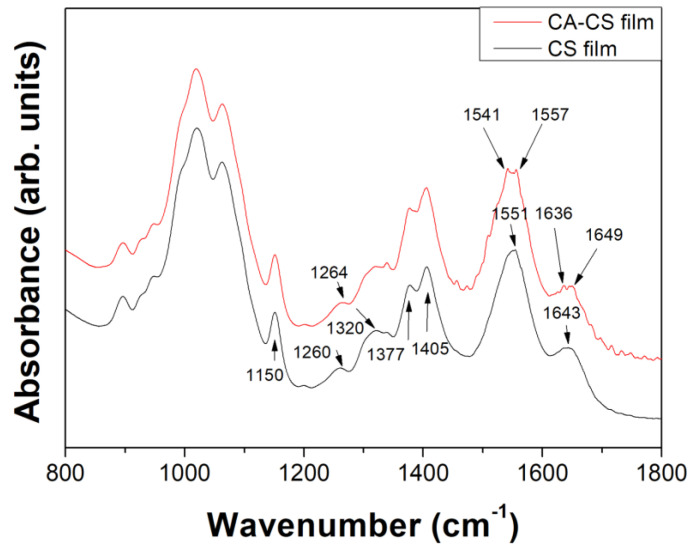
ATR spectra of a neat chitosan film (CS film) and a mCLEAs-modified chitosan film with caffeic acid derivatives (CA-CS film).

To evaluate the changes in chemical bonds of the modified chitosan films, ATR/FTIR analysis was performed. The ATR spectra of the unmodified and modified CS films with caffeic acid in the absence of DES are presented in Figure 10. In the case of the neat CS films, the bands appearing in the range 800–1150 cm^−1^ correspond to C–C and C–O vibrations from the polymer backbone [79]. The characteristic peak of CS at 1150 cm^−1^ arises from anti-symmetric stretching of the C–O–C bonds of the saccharide structure, while the peak at 1320 cm^−1^ corresponds to C-N vibrations [80]. The presence of the band at 1377 cm^−1^ is indicative for the deacetylation degree of CS, since it corresponds to acetamide groups [79]. The peak at 1405 cm^−1^ is assigned to carboxyl groups due to the presence of acetic acid. Finally, the bands at 1551 and 1643 cm^−1^ correspond to N-H and C=O vibrations, respectively [79,80]. 

In the spectrum of caffeic acid-modified CS film the band appearing at 1551 cm^−1^ in the neat CS film was split in two minor peaks at 1541 and 1557 cm^−1^. This change could be associated with the conversion of primary amines into secondary amines during the Michael addition reaction between the -NH groups present in CS and the aromatic and non-aromatic -OH groups of CA [47,81]. In addition, the peak of the neat chitosan at 1643 cm^−1^ disappeared after modification with caffeic acid, while two minor peaks at 1636 and 1649 cm^−1^ appeared. These peaks could be associated with the C-N stretching vibration of imines, indicative of the formation of a Schiff’s base during the enzymatic modification [47,82]. The ATR spectra of the unmodified and mCLEAs-modified Bet:Gly (1:3) -containing CS films, are shown in Figure S6; the presence of DES bands [83] in the spectra of the films confirm the successful employment of the plasticizer. However, since these bands prevail over the characteristic CS bands, no significant changes between the unmodified and the modified DES-containing films could be detected.

**Figure 11:**
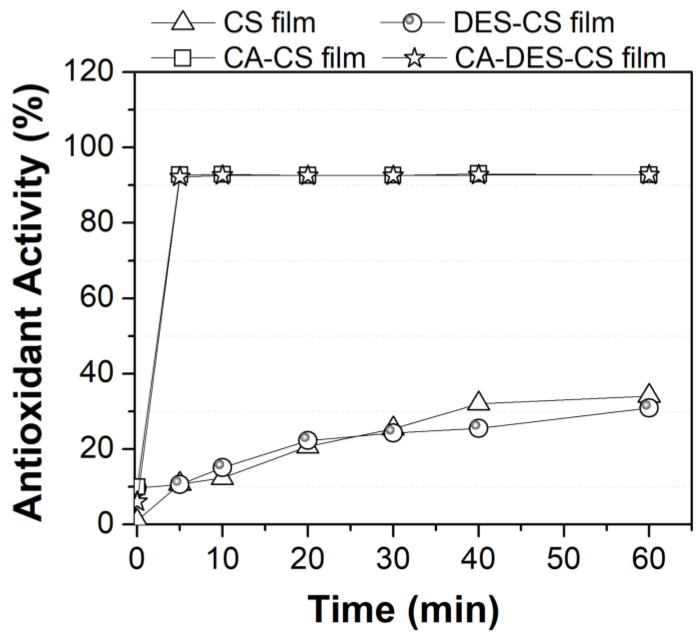
Antioxidant activity of a neat chitosan film (CS film), a chitosan film containing 10% *v*/*v* DES (DES-CS film), a mCLEAs-modified chitosan film with caffeic acid (CA-CS film), and a mCLEAs-modified chitosan film containing 10% *v*/*v* DES with caffeic acid (CA-DES-CS film).

The ABTS radical cation scavenging activity of the unmodified and enzymatically modified CS films is presented in Figure 11. The mCLEAs-modified films, both in the presence and in the absence of DES, exhibited the highest antioxidant activity within the first 5 min of the assay. The antioxidant activity of the neat and the DES-containing CS film was the same, namely ~35% after 1 h, indicating that the presence of the DES had no effect. The innate antioxidant property of the unmodified CS film is generally ascribed to chitosan’s hydroxyl groups and amino groups [42]. The functionalization of CS with several phenolic compounds in their native or oxidized form has been demonstrated to lead to an enhancement in its reducing power [84,85]. This enhancement is attributed to the hydroxyl groups of the phenolic compounds and their oxidation products that can donate a hydrogen atom to stabilize the ABTS radical [78].

## 4. Conclusions

In this work, the biocatalytic and structural properties of tyrosinase were studied in DES aqueous solutions, providing additional insight into the function of oxidoreductases in this class of solvents. Tyrosinase was extracted from mushrooms and magnetic nanoparticles were successfully employed to produce an efficient magnetic biocatalyst that offers the advantage of easy recovery and reusability by implementing a magnetic field. The biocatalyst demonstrated good reusability and excellent storage stability, retaining the 90% of its activity after 5 repeated cycles and no loss of its initial activity after storage at −20 °C for 1 year. The presence of DES in the reaction medium affected the activity of the free and the immobilized enzyme in a different manner, depending on the nature of each DES. Compared to free tyrosinase, the immobilization had a stabilizing effect on tyrosinase in the presence of most DESs. Tyrosinase mCLEAs have successfully implemented in the functionalization of chitosan with caffeic acid in the presence of 10% *v*/*v* Bet:Gly (1:3) for the first time, enhancing the plasticity and antioxidant activity of the chitosan film.

In conclusion, the implementation of DES could enrich the spectrum of biocatalytic applications of free or immobilized tyrosinase in green media at mild reaction conditions.

## Figures and Tables

**Table 1:** DESs prepared in the current work.

DES	Abbreviation	Molar Ratio
Choline Chloride:Urea	ChCl:U	1:2
Choline Chloride:Glycerol	ChCl:Gly	1:2
Choline Chloride:Ethylene Glycol	ChCl:EG	1:2
Choline Chloride:Propylene Glycol	ChCl:PG	1:2
Choline Chloride:Butylene Glycol	ChCl:BG	1:2
Choline Chloride:Butylene Glycol	ChCl:BG	1:4
Choline Chloride:Fructose:H_2_O	ChCl:Fru:H_2_O	5:2:5
Choline Chloride: Urea:Glycerol	ChCl:U:Gly	1:1:1
Choline Chloride:Urea:Ethylene Glycol	ChCl:U:EG	1:1:1
Choline Chloride:Glycerol:Ethylene Glycol	ChCl:Gly:EG	1:1:1
Ethylammonium Chloride:Urea	EAC:U	1:1.5
Ethylammonium Chloride:Glycerol	EAC:Gly	1:1.5
Ethylammonium Chloride:Ethylene Glycol	EAC:EG	1:1.5
Betaine:Glycerol	Bet:Gly	1:2
Betaine:Glycerol	Bet:Gly	1:3
Betaine:Ethylene Glycol	Bet:EG	1:3
Choline Dihydrogen Phosphate:Glycerol	Chol DHP:Gly	1:3
Choline Dihydrogen Phosphate:Ethylene Glycol	Chol DHP:EG	1:2

**Table 2:** Apparent kinetic parameters of tyrosinase mCLEAs for the oxidation of L-DOPA in 10% *v*/*v* aqueous solutions of DESs with the same HBD.

DES	K_m_^app^ [mM]	V_max_^app^ [μΜ min^−1^]
No DES	9.7 ± 1.4	26.0 ± 1.6
ChCl:Gly	12.5 ± 1.9	20.1 ± 1.5
EAC:Gly	11.0 ± 1.6	13.6 ± 0.9
Bet:Gly (1:3)	9.0 ± 2.0	15.5 ± 1.4
Chol DHP:Gly	27.6 ± 5.2	31.9 ± 3.9

**Table 3:** Apparent kinetic parameters of tyrosinase mCLEAs for the oxidation of L-DOPA in 10% *v*/*v* aqueous solutions of DESs with the same HBA.

DES	K_m_^app^ [mM]	V_max_^app^ [μΜ min^−1^]
No DES	9.7 ± 1.4	26.0 ± 1.6
ChCl:Gly	12.5 ± 1.9	20.1 ± 1.5
ChCl:U	15.2 ± 2.7	16.4 ± 1.5
ChCl:U:Gly	12.1 ± 1.8	15.0 ± 1.1
ChCl:BG (1:4)	22.2 ± 4.2	14.2 ± 1.6
ChCl:Fru:H2O	9.9 ± 0.9	13.0 ± 0.5

**Table 4:** CD spectral secondary structural content of free tyrosinase after 5 min of incubation in various DESs (10% *v*/*v*).

DES Concentration	α-Helix (%)	*β*-Sheet (%)	Random Coil (%)
DES-free	31	16	54
10% *v*/*v* Chol DHP:EG	31	12	57
10% *v*/*v* ChCl:Fru:H2O	29	13	57
10% *v*/*v* ChCl:Gly:EG	28	15	56
10% *v*/*v* ChCl:EG	26	18	56
10% *v*/*v* ChCl:Gly	26	20	54
10% *v*/*v* ChCl:U	25	19	56
10% *v*/*v* ChCl:PG	24	25	51
10% *v*/*v* EAC:EG	22	23	55
10% *v*/*v* ChCl:U:Gly	21	26	53

**Table 5:** CD spectral secondary structural content of free tyrosinase after 24 h of incubation in various DESs (10% *v*/*v*).

DES Concentration	α-Helix (%)	*β*-Sheet (%)	Random Coil (%)
DES-free	17	31	52
10% *v*/*v* ChCl:PG	9	43	48
10% *v*/*v* ChCl:EG	9	43	48
10% *v*/*v* ChCl:U:EG	8	44	48
10% *v*/*v* EAC:EG	5	48	48
10% *v*/*v* ChCl:Fru:H_2_O	4	48	48
10% *v*/*v* Chol DHP:EG	4	48	48

## Data Availability

Not applicable.

## Supplementary Materials

The following supporting information can be downloaded at: https://www.mdpi.com/article/10.3390/biom13040643/s1, Figure S1: Relative activity of tyrosinase CLEAs and tyrosinase mCLEAs. The activity measurements were performed under the same experimental conditions: 1 mg mL^−1^ biocatalyst, 10 mM L-DOPA, 50 mM phosphate buffer pH 7, 700 rpm, 30 °C; (b) Photographic evidence that tyrosinase mCLEAs are magnetic and can be collected by applying a magnetic field; Figure S2: Fluorescence emission spectra of free tyrosinase in increasing concentrations (10, 20, 50% *v*/*v*) of (a) ChCl:Fru:H_2_O; (b) ChCl:BG (1:4); (c) Bet:Gly (1:3); (d) EAC:Gly; (e) ChCl:U:Gly; (f) ChCl:U; (g) Chol DHP:Gly; (h) ChCl:Gly; Figure S3: Far-UV CD spectra of free tyrosinase in the presence of 10% *v*/*v* of various DESs, after 5 min and 24 h of incubation. The spectra of the enzyme in the pure buffer are shown for comparison. The concentration of tyrosinase was 0.012 mg mL^−1^; Figure S4: Photographic evidence of the gelation that occurs in the chitosan solution that contains tyrosinase mCLEAs, chitosan and caffeic acid, due to the oxidative action of the biocatalyst (right). The solutions of chitosan and caffeic acid (left) and chitosan and tyrosinase mCLEAs (middle) do not show the gelation phenomenon; Figure S5: Photographic evidence of the color change (browning) of chitosan during the oxidation-grafting reaction of caffeic acid via the action of tyrosinase mCLEAs. The solution of chitosan and caffeic acid remains yellowish in the course of time; Figure S6: ATR spectra of the neat chitosan film (CS film), the chitosan film containing 10% *v*/*v* DES (DES-CS film), the mCLEAs-modified chitosan film with caffeic acid containing 10% *v*/*v* DES (CA-DES-CS film); the spectrum of DES Bet:Gly (1:3) is shown for comparison. In the DES’s spectrum, the bands observed at 930–1110 cm^−1^ are attributed to glycerol, whereas the bands in the 1331–1624 cm^−1^ can be assigned to betaine [83].
