# Supplementary material for: Tyrosinase Magnetic Cross-Linked Enzyme Aggregates: Biocatalytic Study in Deep Eutectic Solvent Aqueous Solutions
Source: Biomolecules. 2023 Apr 3;13(4):643. doi: 10.3390/biom13040643 (PMC10135570; doi:10.3390/biom13040643)
Supplement: Supplementary file 1 [file biomolecules-13-00643-s001.zip › biomolecules-2307513-supplementary.pdf]

Tyrosinase Magnetic Cross-Linked Enzyme Aggregates: Biocatalytic Study in Deep Eutectic Solvent Aqueous Solutions

Myrto G. Bellou [1], Michaela Patila [1,2], Renia Fotiadou [1], Konstantinos Spyrou [2,3], Feng Yan [4], Petra Rudolf [4], Dimitrios P. Gournis [2,3] and Haralambos Stamatis [1,2,*]

[1] Biotechnology Laboratory, Department of Biological Applications and Technologies, University of Ioannina, 45110, Ioannina, Greece;
[2] Nanomedicine and Nanobiotechnology Research Group, University of Ioannina, 45110, Ioannina, Greece;
[3] Ceramics and Composites Laboratory, Department of Materials Science and Engineering, University of Ioannina, 45110, Ioannina, Greece;
[4] Zernike Institute for Advanced Materials, University of Groningen, Nijenborgh 4, 9747 AG, Groningen, the Netherlands
* Correspondence: hstamati@uoi.gr (H.S.)

SUPPLEMENTARY MATERIAL

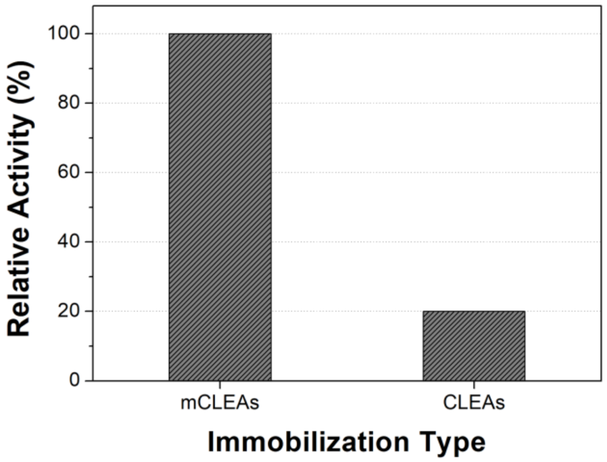

(a)

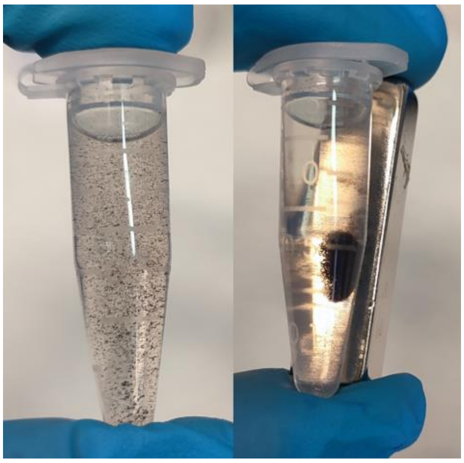

(b)

Figure S1. (**a**) Relative activity of tyrosinase CLEAs and tyrosinase mCLEAs. The activity measurements were performed under the same experimental conditions: 1 mg mL^{-1} biocatalyst, 10 mM L-DOPA, 50 mM phosphate buffer pH 7, 700 rpm, 30 °C; (**b**) Photographic evidence that tyrosinase mCLEAs are magnetic and can be collected by applying a magnetic field.

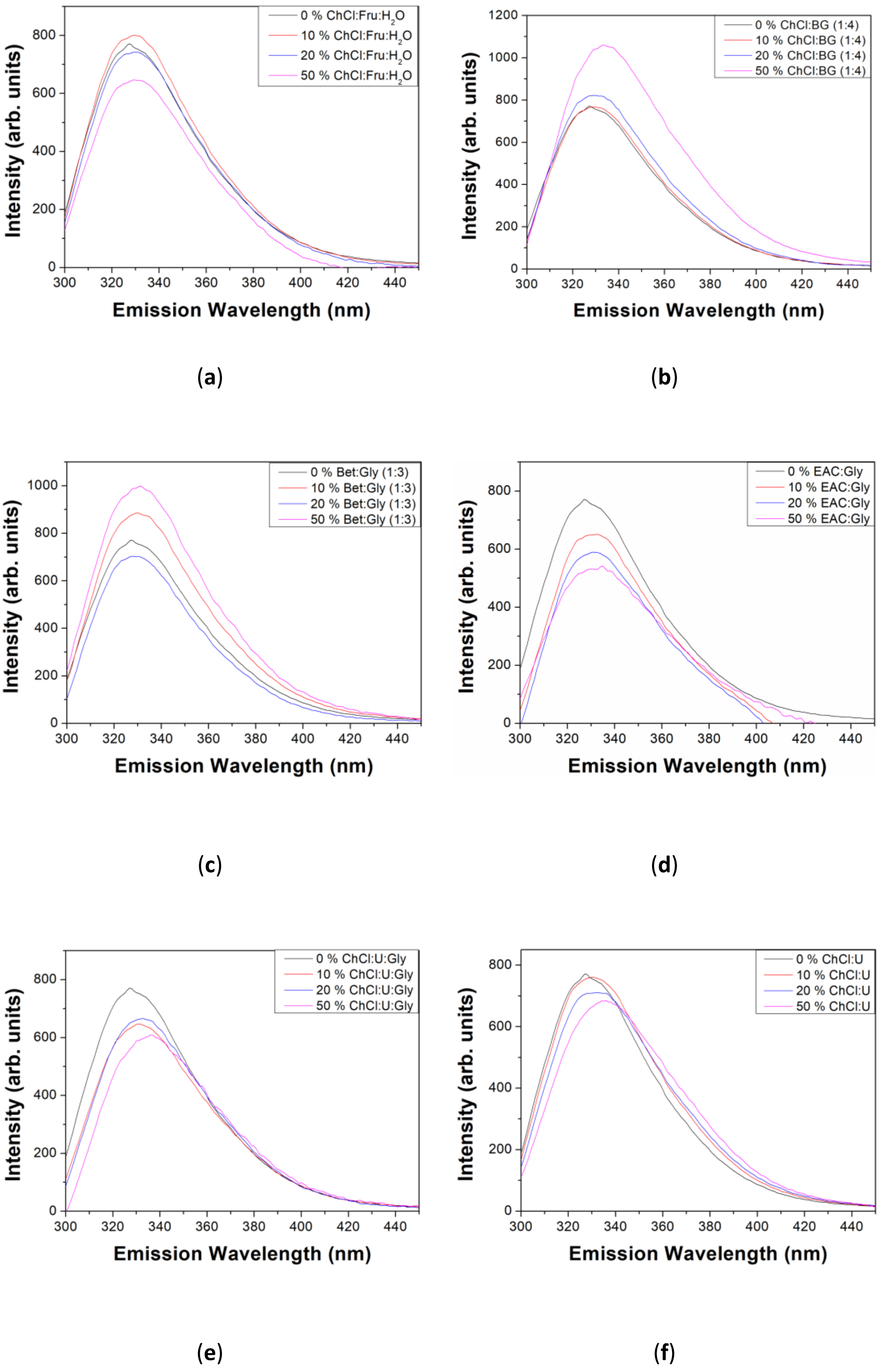

Intensity (arb. units)
Emission Wavelength (nm)
0 % ChCl:Fru:H2O
10 % ChCl:Fru:H2O
20 % ChCl:Fru:H2O
50 % ChCl:Fru:H2O
(a)
0 % ChCl:BG (1:4)
10 % ChCl:BG (1:4)
20 % ChCl:BG (1:4)
50 % ChCl:BG (1:4)
(b)
0 % Bet:Gly (1:3)
10 % Bet:Gly (1:3)
20 % Bet:Gly (1:3)
50 % Bet:Gly (1:3)
(c)
0 % EAC:Gly
10 % EAC:Gly
20 % EAC:Gly
50 % EAC:Gly
(d)
0 % ChCl:U:Gly
10 % ChCl:U:Gly
20 % ChCl:U:Gly
50 % ChCl:U:Gly
(e)
0 % ChCl:U
10 % ChCl:U
20 % ChCl:U
50 % ChCl:U
(f)

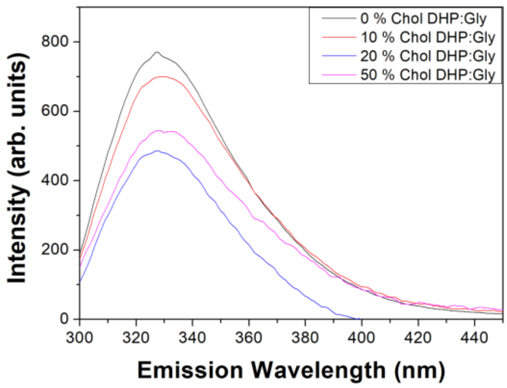

(g)

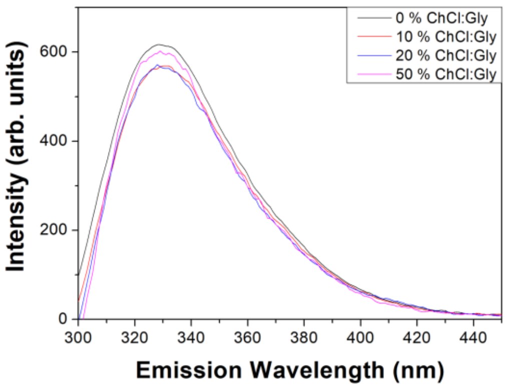

(h)

Figure S2. Fluorescence emission spectra of free tyrosinase in increasing concentrations (10, 20, 50 % *v*/*v*) of (**a**) ChCl:Fru:H_2O; (**b**) ChCl:BG (1:4); (**c**) Bet:Gly (1:3); (**d**) EAC:Gly; (**e**) ChCl:U:Gly; (**f**) ChCl:U; (**g**) Chol DHP:Gly; (**h**) ChCl:Gly.

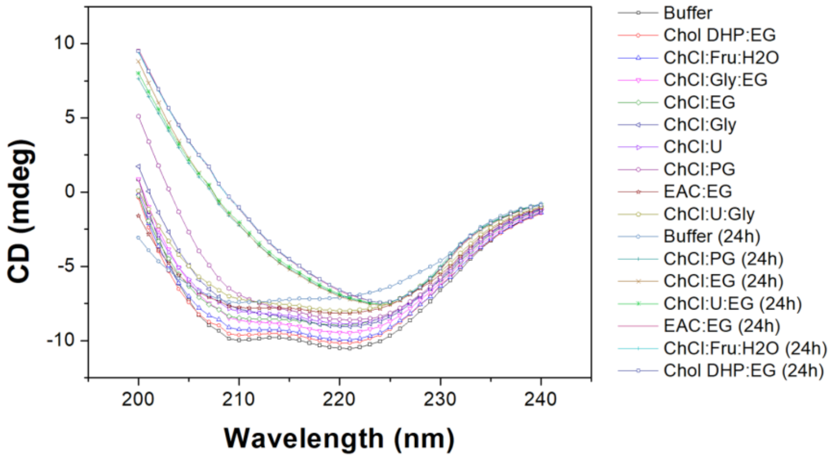

Figure S3. Far-UV CD spectra of free tyrosinase in the presence of 10 % *v*/*v* of various DESs, after 5 min and 24 h of incubation. The spectra of the enzyme in the pure buffer are shown for comparison. The concentration of tyrosinase was 0.012 mg mL^{-1}.

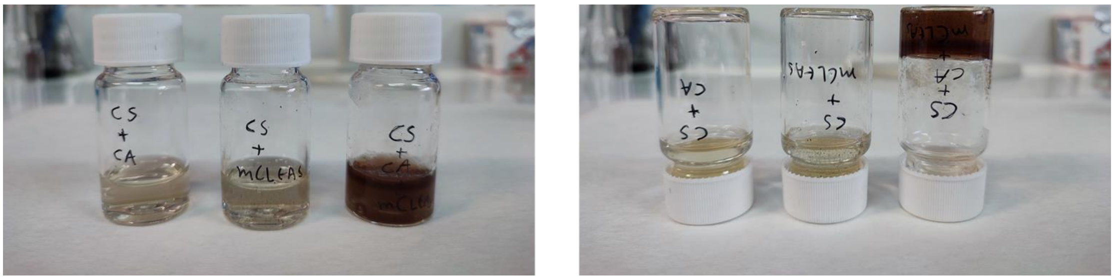

Figure S4. Photographic evidence of the gelation that occurs in the chitosan solution that contains tyrosinase mCLEAs, chitosan and caffeic acid, due to the oxidative action of the biocatalyst (right). The solutions of chitosan and caffeic acid (left) and chitosan and tyrosinase mCLEAs (middle) do not show the gelation phenomenon.

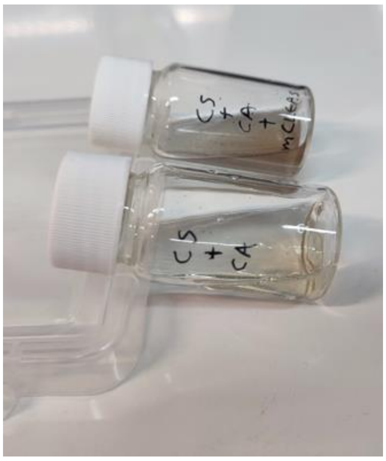

Figure S5. Photographic evidence of the color change (browning) of chitosan during the oxidation-grafting reaction of caffeic acid *via* the action of tyrosinase mCLEAs. The solution of chitosan and caffeic acid remains yellowish in the course of time.

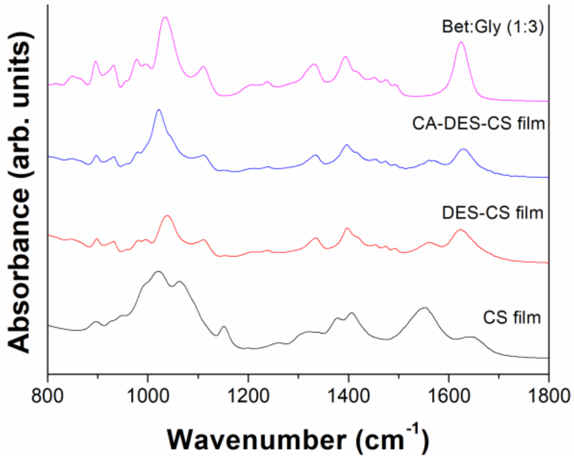

Figure S6. ATR spectra of the neat chitosan film (CS film), the chitosan film containing 10 % *v/v* DES (DES-CS film), the mCLEAs-modified chitosan film with caffeic acid containing 10 % *v/v* DES (CA-DES-CS film); the spectrum of DES Bet:Gly (1:3) is shown for comparison. In the DES's spectrum, the bands observed at 930-1110 cm^{-1} are attributed to glycerol, whereas the bands in the 1331-1624 cm^{-1} can be assigned to betaine [83].
